# Analysis of antibacterial and antibiofilm activity of purified recombinant Azurin from *Pseudomonas aeruginosa*

**Published:** 2019-04

**Authors:** Hajar Mohammadi-Barzelighi, Bahram Nasr-Esfahani, Bita Bakhshi, Bahram Daraei, Sharareh Moghim, Hossein Fazeli

**Affiliations:** 1 Department of Microbiology, School of Medicine, Isfahan University of Medical Sciences, Isfahan, Iran; 2 Department of Bacteriology, Faculty of Medical Sciences, Tarbiat Modares University, Tehran, Iran; 3 Toxicology and Pharmacology, School of Pharmacy, Shahid Beheshti University of Medical Sciences, Tehran, Iran

**Keywords:** Antibacterial effect, Antibiofilm activity, Recombinant Azurin

## Abstract

**Background and Objectives:**

The aim of this study was to evaluate the antibacterial and antibiofilm activity of recombinant Azurin from *Pseudomonas aeruginosa* against different bacterial species.

**Materials and Methods:**

The *azurin* gene was cloned in the pET21a vector. The pET21a-*azurin* construct was transformed into *Escherichia coli* BL21. The recombinant Azurin was expressed and purified using affinity chromatography and confirmed by Western blotting. The cytotoxicity of rAzurin was assessed on peripheral blood mononuclear cells. Antibacterial and antibiofilm activity of rAzurin with different concentrations were determined by micro-broth dilution and crystal violet methods, respectively. The effect of rAzurin on bacterial species was statistically analyzed by t-test and spearman correlation.

**Results:**

The identity of purified protein was confirmed by blotting and distinguished as a 14 kDa band on 15% SDS-PAGE. The IC50 of rAzurin on Peripheral Blood Mononuclear Cell (PBMC) was determined as 377.91±0.5 μg/mL in 24 h. *Vibrio cholerae* and *Campilobacter jejuni* displayed the most sensitivity to rAzurin (27.5 and 55 μg/mL, respectively) and the highest resistance (220 μg/mL) was displayed by *P. aeruginosa* and *E. coli.* The MIC for other species was 110 μg/mL. The Minimum Biofilm Inhibition Concentration (MBIC) was determined as 220 μg/mL for *Salmonella enterica* and *V. cholerae*, 300 μg/mL for *Shigella sonnei, Shigella flexneri* and *P. aeruginosa* and 440 μg/mL for the other species. The antimicrobial effect of rAzurin on bacterial species were significant (p value<0.05) and correlation coefficient was negative.

**Conclusion:**

The rAzurin appears to be an appropriate choice and a new strategy for prevention of bacterial infection. It inhibits bacterial growth and biofilm formation and candidates as antimicrobial peptides.

## INTRODUCTION

Antibiotic resistance is a public health concern and is an obvious outcome of the bacterial adaptation to antibacterial agents (1). Indeed, there is a race between humans for developing new antibacterial agents and bacteria for evolving new resistance mechanisms (2). Approaches for solving this concern include: controlling of antibiotic consumption, better understanding of the resistance mechanisms and expanding new drugs for replacing antibiotics (3).

Multidrug-resistant (MDR) bacteria which are isolated from clinical settings have a potential for creating biofilms and increasing the possibility of progression into chronic, persistent and recurrent infections (4). The biofilm lifestyle permits the bacteria to survive in aggressive conditions such as high concentrations of antibiotics, immune response and capacity to persist in the host body (5). The bacteria that grow in biofilms mainly display increased resistance to all antibiotics. So, we urgently need to develop new and alternative strategies for eradication of biofilm infections in medical settings (6). Bacterial attachment to surfaces is mediated by numerous factors such as adhesion surface proteins such as pili or fimbriae and specific exopolysaccharides (7, 8). The adhesion process occurs most easily on surfaces which are rough, hydrophobic, and coated with surface films (9). Hence, an effective strategy for preventing biofilm formation is inhibition of bacterial attachment and aggregation to the surfaces (4). A novel approach for preventing and eradicating biofilm is using agents that interfere with the structure of biofilm and have excessive potential in the controlling of biofilm-mediated infections. These new strategies include using small molecules, enzyme treatments that decline the biofilm structure, immunotherapy, Quorum-sensing inhibitors (QSI), bacteriophage therapy, signal transduction interference and vaccines that target important phases of biofilm formation (4).

Antimicrobial peptides (AMPs) offer new potential for eliminating infectious diseases. These peptides inhibit the growth of microorganisms and have a broad antimicrobial activity without any effects on the host which produced them. AMPs are part of the innate immune response that is found among all classes of life (10). The bacteriocins as a group of AMPs are produced by bacteria and induced by stress (10).

Azurin is a low molecular weight, blue, copper-containing protein that acts in respiratory electron transport chain in some bacteria (11). In *Pseudomonas aeruginosa*, the periplasmic Azurin is necessary for bacterial protection from oxidative stress (electron donor to nitrate reductase) and copper toxicity. The Azurin properties such as structure, physicochemical and potential to bind different proteins categorize it as an important natural scaffold protein which can be used for different purposes such as anti-tumor, anti-parasite and anti-HIV agent (12, 13).

The purpose of this study was the evaluation of the antibacterial and antibiofilm effect of recombinant Azurin (rAzurin) against different bacteria such as *Staphylococcus aureus, Salmonella enterica* Typhi, *Salmonella* Typhimurium, *Shigella sonnei, Shigella flexneri, Campylobacter jejuni, Vibrio cholerae, Escherichia coli* and *Pseudomonas aeruginosa.*

## MATERIALS AND METHODS

### Bacterial strains.

Bacterial strains include *S. aureus* ATCC25923, *E. coli* ATCC25922, *P. aeruginosa* ATCC25853, *V. cholerae* ATCC14035, *S. flexneri* ATCC12022, *S. sonnei* ATCC25931, *S.* Typhi PTCC1609, *S. enterica* Typhimurium ATCC14028 and *C. jejuni* ATCC29428. The bacteria were primarily streaked on Brain Heart Infusion (BHI) agar and sub-cultured on Mannitol Salt Agar, Eosin Methylene Blue Agar (EMB), MacConkey agar (Mac), Thiosulfate Citrate Bile Salts Sucrose Agar (TCBS), Salmonella-Shigella agar (SS) and Modified Charcoal-Cefoperazone-Deoxycholate Agar (mC-CDA) regarding to *S. aureus, E. coli, P. aeruginosa, V. cholerae, Shigella* and *Salmonella* spp, and *C. jejuni*, respectively. All culture media were purchased from Ibresco, Iran. A single colony from each bacterial strain was grown in BHI broth (Ibresco, Iran) for 24 h at 37°C in shaker to plot the growth curves. For this purpose, the optical density (OD) was measured at 625 nm in 2 h intervals within incubation time and simultaneously, 10 μL of broth culture was serially diluted, cultured on BHI agar, incubated at 37°C for overnight and enumerate the colony forming unit (CFU)/mL.

### Preparation of the rAzurin.

The *azurin* gene was amplified by forward primer Azu-*Nde*I: 5′-GCTCATATGCTACGTAAACTCGCTGCGG-3′ and reverse primer Azu-*Bam*HI: 5′-GACGGATCCATCAGGG TCAGGGTGCCCTT-3′. The primers were designed based on *P. aeruginosa* PAO1 sequence (Gene ID: 878046 and Accession Number: AE004091.2) which encompass the whole sequence of *azurin* gene and harbor the restriction sites of *Nde*I and *Bam*HI enzymes, respectively. The PCR was performed with initial denaturation (95°C, 5 min), 30 cycles of denaturation (95°C, 1 min), annealing (59 for 30 s), extension (72°C, 30 s), and final extension (72°C, 5 min). The products were electrophoresed on 1% agarose gel, and purified by GeneAll Expin Combo GP kit (GeneAll Biotechnology Co., Seoul, Korea) and subjected for direct sequencing using Applied Biosystems (ABI) capillary sequencer (Macrogen, Korea). The similarity of obtained sequence to known sequences was analyzed by Chromas (Technelysium, South Brisbane, Australia), CLC Sequence Viewer 7 (Qiagene, Aarhus, Denmark), and Gene Runner 6.5.46 (Softpedia, Romania) softwares (14–16).

The cloning of *azurin* gene in pET21a vector was performed by double digestion of purified PCR product by 10 U/L *Bam*HI-*Nde*I, (Thermo Fisher Scientific, New York, USA). The pET21a vector was also double digested with the same restriction enzymes. The digested fragments were purified using Gene-All gel Extraction kit (GeneAll Biotechnology Co, Seoul, Korea) and DNA-spin TM plasmid DNA purification Kit (iNtRON biothechnology Co, Seoul, Korea), respectively as manufacturer protocols and incubated at 37°C for overnight with T7 ligation enzyme (Thermo Fisher Scientific, New York, USA). The *azu*-pET21a construct was transformed into the *E. coli* BL21 competent cells. The transformations were confirmed by colony PCR and sequenced using the T7 universal primer (14–16).

The transformed *E. coli* BL21 contain pET21a-*azu* construct was subjected to protein expression and purification. The condition (temperature, time, shaking rate and isopropyl-1-thio-β-D-galactopyranoside (IPTG) concentration) for rAzurin expression was optimized and determined. Briefly, a transformed colony was inoculated into Luria-Bertani (LB) broth (Thermo Fisher scientific, USA) containing 100 μg/mL ampicillin and incubated until reaching the optical density of 0.5–2 at 600 nm. The expression was induced by addition of IPTG 0.5–1 mM to culture media and incubated for 4–6 h. The samples were obtained in 2 h intervals then centrifuged at 4,000 rpm for 20 min. The pellets were mixed with a loading buffer and heated at 95°C for 5 min, and the supernatants were analyzed by SDS-PAGE 15% after centrifugation at 12,000 rpm for 30 min.

The expressed soluble protein was purified from mixed-phased expressions by metal affinity chromatography (Bio-Rad, California, USA) according to the manufacturer's protocol. The BCA protein assay kit (Thermo Fisher scientific, New York, USA) was applied to determine the purified protein concentration. The identity of the purified rAzurin was analyzed by 15% SDS-PAGE and western blotting (WB) assays. Accordingly, the SDS-PAGE gels were blotted onto polyvinylidene difluoride (PVDF) membrane and blocked with 1% skim milk in PBS at 4°C for 18 h. Then, membranes were deepened in anti-polyHistidine (Abcam, Cambridge, USA) at 4°C for 24 h, afterwards incubated with horseradish peroxidase (HRP) conjugated rabbit anti-IgG (Abcam, Cambridge, USA), for 1 h at room temperature and the photoluminescence was recorded by an ECL chemoluminescence imaging system.

### Cytotoxicity assay of rAzurin on peripheral blood mononuclear cell (PBMC).

PBMCs were separated from healthy volunteer's whole blood by Ficoll-Hypaque centrifugation method (400 g, 20 min) (17, 18) and were collected from the interface. The isolated PBMCs were washed 2 times with PBS and purified PBMC were added in Dulbecco's Modified Eagle's Minimal Essential Medium (DMEM) medium (Gibco, Thermo scientific, USA) supplemented with 10% (V/V) inactive fetal bovine serum (Gibco, Thermo scientific, USA) and 1% penicillin-streptomycin. The viable PBMCs were counted by Trypan blue (Sigma-Aldrich, Germany) and seeded in microplate 96-well (SPL, Korea) by inoculating 10^5^ cells/well (19). Afterwards, isolated PBMCs were treated with different concentrations of rAzurin (0–400 μg/mL) for 24 h in humid atmosphere with 5% CO_2_.

For cytotoxicity assay, dimethylthiazol tetrazolium bromide (MTT) (Sigma-Aldrich, Germany) was added to each well and incubated at 37°C for 4 h. The formazan crystals were dissolved by dimethyl sulfoxide (DMSO) (Sigma-Aldrich, Germany) and spectrophotometrically read at 570 nm using a 96-well BioTek Elx808 plate reader (BioTek Instruments, USA). The MTT assay for each concentration of rAzurin was performed triplicate, untreated cells were used as a control and IC50 was also calculated.

### Antibacterial activity of rAzurin.

The Minimal Inhibitory Concentration (MIC) was determined by micro-broth dilution method according to the approved standard by Clinical and Laboratory Standards Institute (CLSI/NCCLS) (2). The overnight bacterial cultures were inoculated to fresh medium and incubated at 37°C to reach the exponential phase base on growth curve which plotted. The bacterial suspensions were adjusted to a density of 1 × 10^6^ CFU/mL and subsequently were added (100 μl) to sterile 96-well microtiter plates (SPL, Korea) (final inoculum size is approximately 5 × 10^5^ CFU/mL) which were treated with different rAzurin concentrations (440, 220, 110, 55, 27.5, 13.75, 6.87, 3.43, 1.71, 0.85, 0 μg/mL) and incubated for 24–48 h (according to bacterial species) at 37°C, each plate also includes a positive growth control and a negative control (sterile control). The MIC of rAzurin was determined as the minimum amount of recombinant protein which capable to inhibit the visible growth of the microorganisms (20). The absorbance of bacterial species in the presence of different concentration of rAzurin was determined in 625 nm. In addition, standard antibiotics from different classes (gentamicin, colistin, trimethoprim and ceftazidime) were included for the evaluation and comparison of the antimicrobial activity.

### Antibiofilm activity of rAzurin.

The *in vitro* biofilm formation assay was carried out using a 96-well microtiter plate as previously described (21–22). Briefly, overnight cultures of bacteria were diluted 1:100 into Mueller Hinton Broth (MHB) and supplemented with 2% w/v sucrose (Sigma-Aldrich, Germany), in the presence and absent of different concentration of rAzurin (0.39–800 μg/mL). The microplate was incubated at 37°C for 24–48 h under static conditions. After incubation, the established biofilm was washed twice with 200 μL of distilled water, dried and stained with 100 μL of 0.4% crystal violet (Merck, Germany) for 30–45 min. Afterwards, the wells were washed four times with distilled water and discolored with 200 μL of ethanol 95% (Merck, Germany) for 45 min, 100 μL of the discolored solution was transferred into the well of a new plate and the Crystal Violet was measured at 570 nm in a microplate reader (BioTek, Elx808, USA). The absorbance value in treated and untreated wells were compared. This assay was performed in triplicate in at least three individual experiments for each concentration (22). The Minimum Biofilm Inhibition Concentration (MBIC) was defined as the minimal anti-microbial concentration at which there was no observable bacterial growth in wells containing adherent bacteria.

### Statistical analysis.

All experiments were performed in triplicate in three different assays. Results were expressed as mean ± SD. The differences of absorbance and growth between rAzurin-treated and control wells were compared using the t-test by considering of inhibitory concentration as breakpoint in antibacterial and antibiofilm assays and the P-values of < 0.05 were considered as significant. The correlation between rAzurin concentration and growth rate or biofilm mass development were analyzed by spearman correlation.

## RESULTS

### Bacterial strains.

The growth curve of bacteria was plotted (data not shown) and the optical density for *S. aureus, E. coli, P. aeruginosa, V. cholerae, Shigella* spp., *Salmonella* spp. and *C. jejuni* in bacterial population of 10^8^ CFU/mL were determined 1, 1.1, 1.3, 0.9, 1.1, 1 and 0.9, respectively.

### Preparation of the rAzurin.

**Fig. 1.**
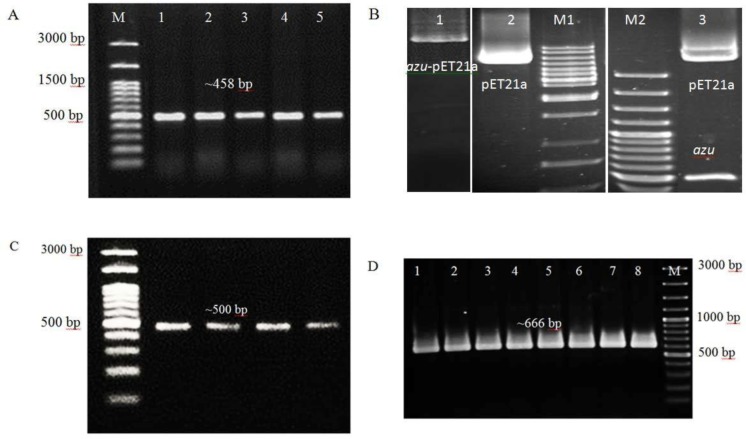
(A) Agarose gel electrophoresis represents amplification of *azurin* gene M: DNA molecular weight 100 bp; (B) Digested and purified pET21a-*azu* construct and pET21a by *BamH*1 in line 1, and line 2, respectively, double digested of purified pET21a-*azu* construct by *BamH*1 and *Nde*1 in line 3, M1: molecular marker 1kb, M2: molecular marker 100 bp. C, Purified *azurin* fragments were double digested using *NdeI* and *BamHI*, M: molecular marker 100 bp, lines 1–4; (D) Colony PCR amplification of *azurin*-pET21a construct by T7 specific primers in lines 1–8, M: molecular marker 100 bp.

The PCR assay of *azurin* gene displays a 458 bp band (Fig. 1A). The product sequence analysis indicated significant similarity to *azurin* gene which is registered in Gene bank. The purified pET21a and *azurin* amplicon were digested and run on 1% agarose gel (Fig. 1B and 1C). The colony PCR assay was performed by T7 primer pairs for approving of the transformation of ligated construct into the competent (Fig. 1D).

**Fig. 2.**
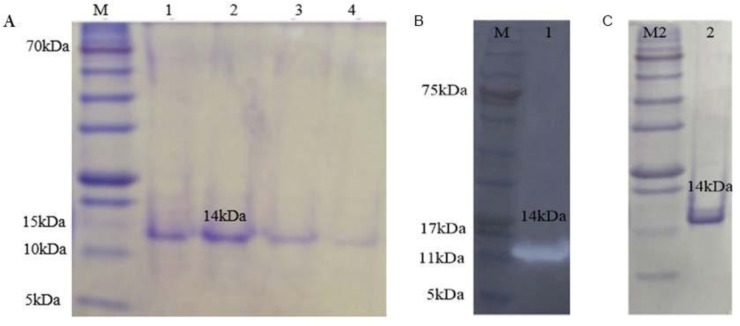
(A) Lines 1–4: Azurin elusion1-4. M: Protein molecular marker; (B) Lines 1 and 2, purified Azurin that visualized by blotting and Commassiee blue, respectively. M: protein molecular marker.

The transformed bacteria were grown on LB broth containing ampicillin (100 μg/mL) and protein was expressed as terminal His-tagged protein. Fig. 2A shows the SDS-PAGE of proteins extracted from *E. coli* BL21. The optimal conditions of protein expression included 1–1.5 optical density, IPTG: 0.5 mM, time: 18 h, temperature: 25°C and terrific broth medium. Purification of soluble rAzurin was performed by Ni-chromatography and monitored in different elution through SDS-PAGE 15%. Fig. 2B shows that purified recombinant protein as 14 KDa band which confirmed by western blotting (Fig. 2C).

### Cytotoxicity assay of rAzurin on peripheral blood mononuclear cell.

The results of the rAzurin cytotoxicity assay indicates that IC50 for rAzurin on PBMCs was 377.91±0.5 μg/mL in 24 h. The viability index of PBMC which treated with 25, 50, 100, 200 and 400 μg/mL of rAzurin, was about 88.8, 81.6, 75.2, 64.8 and 49.6%, respectively.

### Minimal inhibitory concentration (MIC) for rAzurin.

**Fig. 3.**
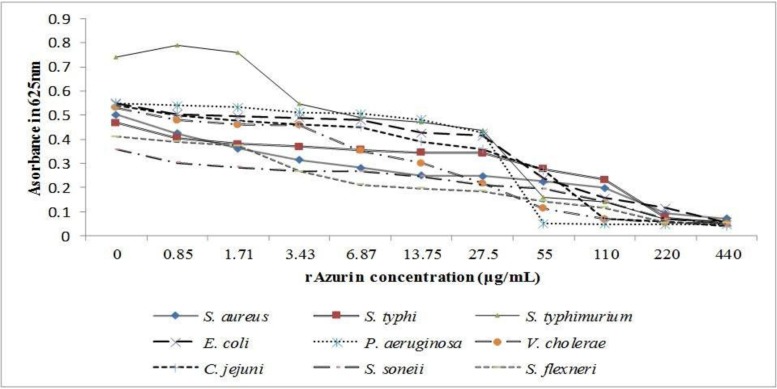
The effect of rAzurin concentrations (0.85–440 μg/mL) on the growth of different bacterial species. The growth rate of bacterial species decrease by increasing of rAzurin concentrations. The absorbance of bacterial species in the presence of different concentration of rAzurin were significantly different (p<0.01) by considering MIC as breakpoint.

Antimicrobial activity of rAzurin versus *S. aureus, S.* Typhi, *S.* Typhimurium, *S. sonnei, S. flexneri, C. jejuni, V. cholerae, E. coli* and *P. aeruginosa* were depicted in Fig. 3. As were presented in Fig. 3, the absorbance of bacterial mass at 625 nm decreased by increasing of rAzurin concentration in a dose-dependent manner. The results also revealed reduction of bacterial CFU/mL with increasing rAzurin concentrations and the correlation coefficient was negative for all species which studied. The MIC of rAzurin for different bacterial species is displayed in Table 1, the lowest MIC belonged to *V. cholerae* (27.5 μg/mL), whereas *P. aeruginosa* and *E. coli* were the most resistant species (220 μg/mL). The MIC in the remaining species were reported 110 μg/mL. The absorbance of all bacterial species in the presence of rAzurin were compared between the wells containing the higher and lower concentrations of rAzurin than MIC by t-test and the difference (p<0.05) was considered as significant.

**Table 1.** Minimum inhibitory concentration (MIC) and minimum biofilm inhibition concentration (MBIC) of rAzurin (μg/mL) and MIC of standard antibiotics against different bacterial species.

**Bacterial species**	**MIC (μg/mL)**					**MBIC of rAzurin (μg/mL)**

	**rAzurin**	**Gentamicin**	**Colistin**	**Trimethoprim**	**Ceftazidime**	
*S. aureus* ATCC25923	110	≤0.5	>16	2	2	440
*S. enterica subsp. enterica* serovar *typhi* PTCC1609	110	1	2	≤0.25	<0.5	220
*S. enterica subsp. enterica* serovar Typhimurium ATCC14028	110	1	2	≤0.25	0.5	220
*P. aeruginosa* ATCC25853	220	2	24	>128	4	300
*C. jejuni* ATCC29428	55	≤0.5	16	>32	>8	440
*S. sonnei* ATCC25931	110	2	2	≤0.25	<0.5	300
*S. flexneri* ATCC12022	110	2	2	<0.5	0.5	300
*V. cholerae* ATCC14035	27.	5				220
*E. coli* ATCC25922	220	1	≤1	1	≤0.5	440

### Antibiofilm activity.

**Fig. 4.**
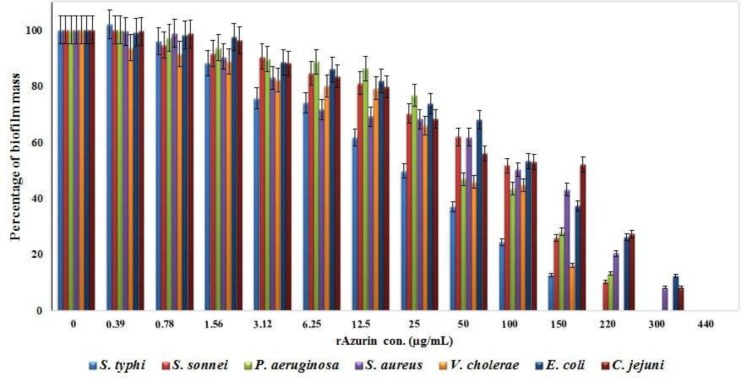
Percentage of biofilm mass (Biofilm adsorbance in the presence of rAzurin/biofilm adsorbance without rAzurin)×100 in the presence of different concentrations of rAzurin (μg/mL) and studied bacterial species which were specified by different colors. The biofilm mass percentage in all bacterial species reduced by incresing of rAzurin concentrations. The reduction rate in MBIC concentrations compared to control well were significant (p<0.05).

We analyzed the ability of bacterial strains to form biofilm in the presence of different concentration of rAzurin (0–800 μg/mL). The biofilm growth and development were measured by assessing the biofilm mass using Crystal Violet staining. The absorbance of bacterial biofilms is presented in Fig. 4. As were shown in Fig. 4, the biofilm mass in the bacterial species decreased by increasing rAzurin concentration and displayed the negative correlation (correlation coefficient). The MBIC was determined 220 μg/mL for *S.* Typhi, *S.* Typhimurium and *V. cholerae*, 300 μg/mL for *S. sonnei, S. flexneri* and *P. aeruginosa* and 440 μg/mL for the remaining species. The t-test analysis revealed that the biofilm mass reduction was significantly affected by rAzurin concentrations (p<0.05). The MIC of standard antibiotic were displayed in Table 1.

## DISCUSSION

The emergence of antibiotic resistance in bacteria is a major issue for overcoming infectious diseases. The spread of multi-drug resistant bacteria is not only through nosocomial infections but also takes place in the community (23). Recently, an alternative strategy to overcome MDR bacterial infections is the discovery of new antimicrobial agents from various sources. These new antimicrobial elements include human defensins, cathelicidins, and AMPs from bacteria, viruses, plants, vertebrates and invertebrates which possess the potential to replace traditional antibiotics (24–27) and recently has gained increasing attention. The main purpose of this study was to evaluate the antibacterial and antibiofilm activity of the periplasmic protein of *P. aeruginosa*.

The results of antimicrobial assay demonstrated that rAzurin inhibits the growth of most species at 110 μg/mL (Fig. 3., Table 1). *V. cholerae* and *C. jejuni* were the most susceptible species to rAzurin, on the contrary, *E. coli* and *P. aeruginosa* were inhibited at a higher concentration (220 μg/mL) (Fig. 3., Table 1). Since Azurin is produced by *P. aeruginosa*, its low sensitivity is obvious. Then, we supposed this protein acts selectively. The same MIC for *S. aureus* and *Shigella-Salmonella* species (from Gram positive and Gram negative bacteria) may relate to the inhitory mechanism of rAzurin. The inhibitory concentration of standard antibiotics revealed that our results for rAzurin antibacterial effect are reliable and higher concentration of rAzurin compared to standard antibiotics is necessary to inhibit the growth of the same bacteria.

It is demonstrated that amino acids (aa) 50 to 67 of Azurin contain putative protein transduction domain (PTD) which is involved in the interaction with unknown N-glycosylated cell surface protein in eukaryotic cells and is responsible for the penetration of peptide into cancer cells (27–30). Then, we supposed that rAzurin, similar to its interaction with mammalian cell membrane, may interact with proteins or glycosylated proteins on the bacterial cell surface and effect on their function.

Leuschner et al. (2004) demonstrated that amphipathic cytolytic peptides disrupt the cancer cell membrane, mitochondrial permeability and act through a specific receptor-mediated mechanism (30). Considering the structural similarity of Azurin with mentioned amphipathic peptides and the homology between bacteria and mitochondria (31), we assumed that Azurin shows the antibacterial effect by changing the permeability of the bacterial membrane. Of course, the mechanism of Azurin function as antibacterial agent is not clearly known and needs to be more investigated.

It was established that cationic antimicrobial peptides can form α-helix or β-sheet structures for penetration to bacterial cell membrane (32). They also can cross the outer membrane of Gram-negative and the cell wall of Gram-positive bacteria, accumulate them and probably bind to outer membrane proteins and activate lysozyme and damage cell wall (33). AMPs may also act intracellularly and prevent protein folding, enzyme activity, mitochondrial function, protein or DNA/RNA synthesis and show antimicrobial effects (34). Due to structural similarity of Azurin to AMPs which contains α-helix and β-sheet, we supposed that rAzurin may interact with the bacterial cell membrane, influence on its integrity and induce antibacterial activity or penetrates to bacterial cell and regarding to scaffold nature interact with different components and influence on their function.

Involvement of Azurin in antimicrobial activity against parasite and virus has been widely described (12, 13). In several studies determined that Azurin and its derivative can attach to merozoite surface protein 1 (MSP1) of *Plasmodium falciparum*, gp120 of HIV-1, parasite surface antigen SAG1 of *Toxoplasma gondii*, dendritic cell-specific adhesion receptor (DC-SIGN) and intercellular adhesion molecule ICAM-3 then considerably decrease parasitemia, parasite invasion and HIV growth (12, 13).

In our study, inhibitory effect of rAzurin on bacterial growth displayed dose dependent manner wherein the growth of bacterial species reduced by increasing of rAzurin concentration. This confirms that the observed inhibitory effect is completely related to rAzurin.

It has been shown that bacteria could develop biofilms as a persistence strategy and it is a predominant means of growth for microorganisms in natural ecosystems (35). Infectious diseases could be prevented by inhibiting biofilm formation using antiadhesive agents, small molecules, matrix targeting enzymes, immunotherapy, bacteriophage therapy, quorum-sensing inhibitors and signal transduction interface (4, 35). In addition to determining the antibacterial properties, our findings revealed that rAzurin displays a considerable antibiofilm activity against most of the bacterial species were examined in this study.

The effect of rAzurin in preventing biofilm growth was presented in Fig. 4. According to our results, the highest inhibitory effect of rAzurin was observed in *V. cholerae* and *S.* Typhi. The low rAzurin effect on the biofilm formation of *C. jejuni*, despite its significant effect on bacterial growth, may be due to the ineffectiveness of rAzurin on factors which involve in the formation and development of *C. jejuni*, s biofilm.

Since Azurin is a scaffold protein and possess the ability to interact with different proteins (36) and based on the results of our study about the antiadhesive effect of this protein on the attachment of bacteria to Caco2 cell (data have not shown), we supposed that this protein interacts with bacterial adhesins or receptors, thereby inhibits their binding to the surface or to each other. It is also possible that rAzurin may interact with proteins involved in quorum-sensing or signal transduction and causes an overall reduction in biofilm growth by these mechanisms. Of course, the mechanism of Azurin function on bacterial biofilm is not fully known and needs to be further investigated.

Boles et al. (2008) demonstrated that glucose depletion or addition of autoinducing peptides (AIP) in *S. aureus* biofilm, activates *agr* system in established biofilm, leading to disassembly biofilm-associated cells and conversion to a planktonic phenotype (37). In another study has been shown activated the *agr* system can lead to increase of *Staphylococcal* proteases that disrupt cell surface proteins and cell to cell interactions in the biofilm (37). The rAzurin may be function similar to AIPs. The mechanism of quorum-sensing inhibitor (QSI) for inhibition of biofilm growth usually involves suppression of signal generation, blocking of signal receptors and disruption of QS signals (4). We hypothesize that rAzurin may act as QSI by occupying signal receptors.

Park et al. 2007 found that applying an antibody against quorum-sensing peptide AP4 of *S. aureus* have a potency to suppress *S. aureus* pathogenicity in mouse infection model (38). Since the structure of Azurin is similar to Ig superfamily (13), it is supposed that rAzurin may interact with quorum-sensing peptide and interfere with biofilm development.

It has been shown that *P. aeruginosa* produces the medium-chain fatty acid chemical messenger, Cis-2-Decenoic acid (C2DA) which could potentially inhibit biofilm formation in initiation stage, dispersion of existing biofilm of *S. aureus*, other Gram-positive and Gram-negative bacteria (39, 40). As it is seen, *P. aeruginosa* metabolite similar to Azurin inhibits biofilm growth and formation.

In the present study, the antibiofilm property of rAzurin similar to antibacterial activity, showed dose dependent manner in which the biofilm development reduced by increasing of rAzurin concentration.

Our results were displayed, the IC50 of rAzurin on human normal cell (PBMC) was 377.91±0.5 μg/mL in 24 h, which is more than the concentration required to inhibit bacterial growth in all strains and biofilm in some strains assayed in this study. Since the low toxicity is a desirable property for AMPs beside antimicrobial activity, we assumed that identification of epitopes which involved in antibacterial and ant eukaryotic cells is essential.

## CONCLUSION

Given the growing trend of antibiotic resistance, finding compounds that act as an alternative to antibiotics as well as possess antibacterial properties will be valuable. The inhibitory effect of rAzurin on bacterial growth and biofilm formation makes it an appropriate protein for using in many medical settings as a therapeutic agent or a prophylactic approach by preventing biofilm formation and bacterial growth.
